# A Case of Portal Vein Thrombosis in a Patient With Methylenetetrahydrofolate Reductase A1298C Polymorphism

**DOI:** 10.7759/cureus.21743

**Published:** 2022-01-30

**Authors:** Anna Martin, Sierra Struble, Antonio Prado, Jacob Robinson, John Goddard, Travis Smith

**Affiliations:** 1 Osteopathic Medicine, Lake Erie College of Osteopathic Medicine, Bradenton, USA; 2 Emergency Medicine, Lake Erie College of Osteopathic Medicine, Bradenton, USA

**Keywords:** homocysteinemia, prothrombotic state, folate, mthfr, portal vein thrombosis

## Abstract

Portal vein thrombosis (PVT) is a prothrombotic state caused by blood flow stasis, vascular injury, and/or hypercoagulability, resulting in partial or complete occlusion of the portal vein. PVT is a rare diagnosis, particularly among those without liver disease. Typical risk factors for PVT include cirrhosis, hepatocellular carcinoma, myeloproliferative neoplasms, other malignancies, oral contraceptive use, bowel infections, and inherited hypercoagulable disorders. The goal of this study is to analyze a case of PVT in a patient in which no clear etiology could be identified and to evaluate whether the patient’s methylenetetrahydrofolate reductase (MTHFR) polymorphism may have been a risk factor.

This is a case of a 44-year-old female with a history of irritable bowel syndrome, hypertension, hyperlipidemia, sleep apnea, gastric bypass surgery, and MTHFR polymorphism who presented to a walk-in clinic with five days of severe abdominal pain associated with diarrhea, nausea, and anorexia. Hypertension and tenderness over the right lower quadrant prompted a referral to the emergency department for evaluation of possible appendicitis. A contrasted computerized tomography (CT) scan of the abdomen and pelvis revealed a normal appendix and acute portal vein thrombosis. She was then admitted for treatment with intravenous (IV) heparin, fluids, and pain management. After an uneventful three-day hospital course, the patient was discharged on rivaroxaban with a plan to continue anticoagulation therapy for six months and follow up with a hematologist, who later confirmed the patient did not have any inherited hypercoagulable disorders.

It is unclear whether the patient’s MTHFR polymorphism prompted her PVT as existing data on MTHFR’s effects are limited and conflicting. One cannot conclude that MTHFR caused a state of hyperhomocysteinemia to prompt hypercoagulability, as this has not been consistently proven in current literature, and the patient’s homocysteine levels were not measured at the time of diagnosis. This case illustrates that further research on the various MTHFR polymorphisms and their effects on coagulation, potentially via homocysteinemia, is warranted. Further research on the MTHFR polymorphisms may help determine whether providers should test for MTHFR in the evaluation of thrombotic risk factors and may help optimize the treatment of thrombotic events for affected individuals.

## Introduction

Portal vein thrombosis (PVT) is a diagnosis that occurs when the portal vein becomes occluded, partially or completely, by a thrombus [1]. Acute symptoms may include diarrhea, abdominal distention, abdominal pain, vomiting, nausea, fever, and splenomegaly, whereas chronic PVT may present with ascites, splenomegaly, and pancytopenia or be asymptomatic [2]. PVT may be serious and result in death, so it is imperative to begin treatment promptly. A prothrombotic state is the pathophysiological cause of PVT, and risk factors include cirrhosis, hepatocellular carcinoma, myeloproliferative neoplasms, other malignancies, oral contraceptive use, and inherited hypercoagulability [1,2]. Without a history of liver disease, PVT is a rare diagnosis [2]. In this case presentation, the patient did not have a history of liver disease, which prompted further investigation of underlying causes. One possible etiology or risk factor was found to be a previous diagnosis of methylenetetrahydrofolate reductase (MTHFR) polymorphism. Studies have shown that the MTHFR polymorphism can result in reduced activity of methylenetetrahydrofolate reductase resulting in hyperhomocysteinemia [3], and elevated homocysteine may result in a prothrombotic state or serve as a marker for cardiovascular risk [4]. Several studies have hypothesized different mechanisms for the potential prothrombotic state that can result from hyperhomocysteinemia [5]. Nonetheless, data regarding whether or not MTHFR induces a prothrombotic state and whether this is via a setting of hyperhomocysteinemia remains conflicting [6].

## Case presentation

A forty-four-year-old female presented to a walk-in clinic with severe abdominal pain that began five days prior. Associated symptoms included diarrhea, nausea, and anorexia, but she was still passing flatus and having bowel movements. She stated that the pain became more pronounced in her right lower quadrant, which prompted concern for appendicitis. Her past medical history included irritable bowel syndrome, hypertension, hyperlipidemia, obstructive sleep apnea, and anxiety. Her only home medication was escitalopram, which she was taking for anxiety. Prior surgeries included a vertical sleeve gastrectomy and cholecystectomy several years ago, and family history was significant for a transient ischemic attack and a deep vein thrombosis in her mother (who had a history of smoking, obesity, and peripheral vascular disease) and a postpartum cerebrovascular attack in her maternal grandmother.

**Figure 1:**
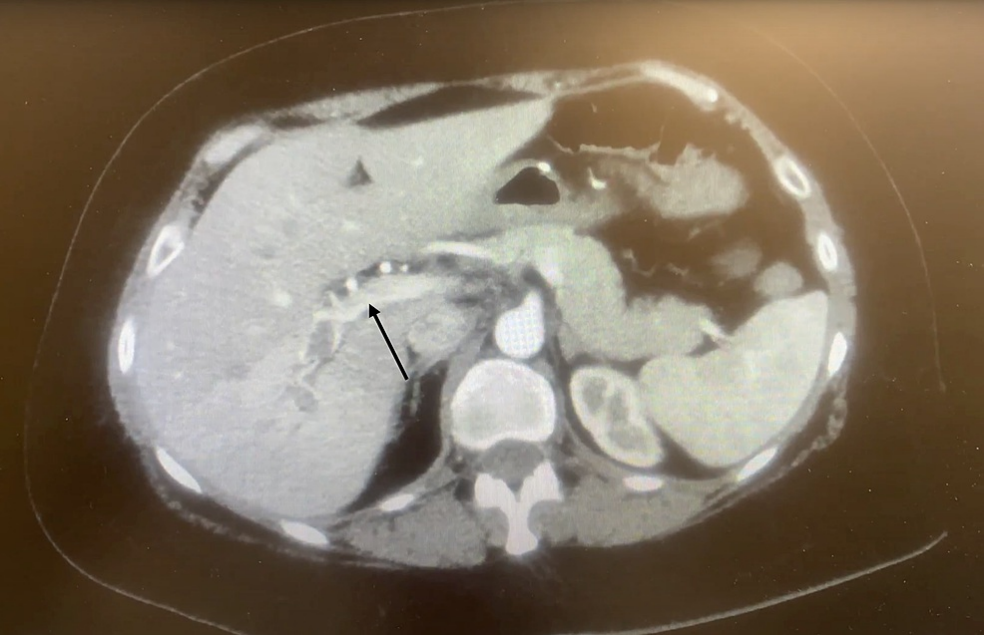
CT scan showing hyperattenuation of the portal vein lumen (arrow) suggestive of PVT and resultant differential enhancement of the liver PVT - portal vein thrombosis

She was advised to go to the emergency department for further work-up of possible appendicitis. Upon arrival, her blood pressure was 162/92 mmHg, respirations were 18 breaths per minute, and oxygen saturation was 98%. She was afebrile. On physical exam, her abdomen was diffusely tender, predominantly in the right lower quadrant. She had no rebound tenderness, guarding, hepatomegaly, splenomegaly, or rectal bleeding. A complete blood count, comprehensive metabolic panel, liver function test, quantitative lipase test, and quantitative lactic acid test were all well within normal limits. A computerized tomography (CT) scan ruled out appendicitis and revealed an acute right portal vein thrombosis that was likely occlusive, with partial thrombosis of the left portal vein and main portal vein (Figure 1). Additionally, a CT scan showed a mild amount of colonic stool without evidence of obstruction, as well as diverticulosis without evidence of acute diverticulitis (not shown). Subsequent coagulation panels were within normal limits, revealing a prothrombin time (PT) of 10.1 sec (N=10-13 sec), activated partial thromboplastin time (APTT) of 22.2 sec (N=30-40 sec), and international normalized ratio (INR) of 1 (N<1.1). Based on clinical and CT findings, anticoagulation with intravenous heparin was started, as well as acetaminophen, morphine, hydrocodone, ondansetron, and IV fluids. The patient was admitted for further treatment and monitoring.

On hospital day two, the patient remained afebrile at 97.7 degrees Fahrenheit, and she reported improved oral intake without vomiting and slightly improved pain but more predominant in the right upper quadrant. The patient was evaluated by general surgery, but no surgical intervention was indicated at this time. The patient continued to pass flatus and normal bowel movements, and her vitals remained within normal limits. Since heparin was initiated, APTT rose from 22.2 sec to 121.3 sec, then ranged from 40.9-51.4 sec throughout the rest of the day. She was assumed to have hypercoagulopathy, but further testing was not ordered at this time as values are typically abnormal during an acute thrombosis. Upon thorough patient questioning regarding potential contributors to her PVT, the patient reported having been previously diagnosed with MTHFR A1298C polymorphism while undergoing genetic testing as part of a customized weight loss plan. This genetic testing report from two years prior also revealed normal homocysteine and folate levels, an elevated fibrinogen level (496 mg/dL; N= 200-400 mg/dL), an elevated C-reactive protein (CRP) (13.8 mg/L; N <10 mg/L), and normal factor V Leiden and prothrombin genotypes.

On hospital day three, the patient continued to be afebrile and reported feeling significantly better, without any right upper quadrant tenderness. Her vitals remained stable, and labs revealed that APTT was still elevated, now at 56.4 sec. INR was not re-tested. Pain and appetite continued to improve, and she was discharged home two days later with oral rivaroxaban 15 mg twice per day (BID). She was advised to remain on anticoagulation for three to six months and see a hematologist for further evaluation of hypercoagulopathy and MTHFR polymorphism. At follow-up, a repeat CT (not shown) confirmed that her thromboses had dissolved, and thus rivaroxaban was stopped. Further work-up for genetic clotting disorders not previously tested in this patient, such as deficiencies of protein C, protein S, or antithrombin, were negative.

## Discussion

The portal vein is formed by the convergence of the splenic vein and superior mesenteric vein, providing drainage for the spleen and small intestine. A portal vein thrombosis (PVT) occurs when a clot partially or completely obstructs the vessel lumen. Because PVT is rare, there is limited data on the incidence in the general population [2], indicating the importance of case analyses. One 2010 multicenter study estimated an incidence rate of 0.7 per 100,000 people and a prevalence of 3.7 per 100,000 in developed countries, with frequency considerably increasing with comorbid cirrhosis or prothrombotic disorders [7]. Data on portal vein thrombosis in individuals without cirrhosis or other liver disorders is particularly limited, illuminating the importance of cases such as this one.

Clinically, PVT presentation depends on the degree of occlusion and acuity. A patient with acute PVT may present with abdominal pain or distention, diarrhea, rectal bleeding, nausea, vomiting, anorexia, fever, lactic acidosis, splenomegaly, and sepsis. In comparison, chronic PVT can be asymptomatic except for varices, cutaneous collaterals, or ascites [2]. In this case, the patient presented acutely with five days of severe right-sided abdominal pain with associated nausea, diarrhea, and anorexia, all of which are typical for acute portal vein thrombosis. Her abdomen was nondistended, and guarding was absent, suggesting the absence of intra-abdominal infection, intestinal infarction, and perforation, all of which are potential complications of PVT. In cases where PVT does lead to bowel ischemia or multiorgan failure, the in-hospital mortality rate is approximately 20%-50% [2]. The PVT in our patient was identified early enough that no intestinal infarction had developed (as confirmed by CT imaging), indicating an overall good prognosis. 

Typically, splenomegaly would suggest the need for ultrasonography, the diagnostic imaging of choice for identifying PVT, due to its high sensitivity and specificity [2]. Because our patient displayed migratory right lower to right upper quadrant tenderness without splenomegaly, providers initially suspected appendicitis, so contrast CT was performed. One advantage of CT imaging over ultrasound (US) imaging is the increased reliability in determining the extent of thrombus to the mesenteric circulation and the effect on the bowel and adjacent organs [2,6]. In this case, the patient’s PVT was not extensive enough to affect her bowels, as the right portal vein was primarily occluded with only partial thrombosis of the left and main portal veins. Further work-up of our patient yielded normal liver tests, as is expected for a patient with PVT without liver disease [2]. APTT was normal, and levels of prothrombin, coagulation factors, and D-dimer were not tested.

To evaluate a PVT, providers should first determine if a patient has cirrhosis, abdominal malignancy, or an abdominal inflammatory focus by performing imaging. Local risk factors such as these account for approximately 70% of PVTs [2]. Once these are ruled out, the standard of care is to evaluate for other risk factors [7,8]. Our patient did not present with symptoms or a history that would indicate cirrhosis of the liver, the risk factor most commonly associated with PVT. CT scans and lab work, including complete blood count (CBC), basic metabolic panel (BMP), a quantitative lipase test, and liver function test, ruled out other common local causes of PVT. It is possible that her history of vertical sleeve gastrectomy and cholecystectomy caused portal venous injury, potentially predisposing her to PVT, but because of the length of time elapsed since these procedures, an iatrogenic cause of PVT is deemed unlikely. 

The other 30% of PVTs without local risk factors are due to inherited or acquired systemic factors, such as myeloproliferative disorders or prothrombotic conditions [2,6]. Among PVT patients with healthy livers, 17-53% have been found to have Philadelphia chromosome-negative myeloproliferative disorders. While abnormal blood counts may suggest this diagnosis, it is also possible for blood counts to be within range, as they were for this patient, likely because PVT’s resultant portal hypertension causes hypersplenism and expanded plasma volume. Partially for this reason, screening for the most prognostic mutation, JAK2 V617F, is recommended [6]. One study found that 19% of PVT patients without malignancy or cirrhosis had JAK2 mutations [7], but screening may also be performed via bone marrow biopsy [6]. Our patient’s prior genetic testing did not include screening for JAK2 V617F, but outpatient work-up with hematology showed the patient was negative for this.

Other common genetic prothrombotic risk factors include factor V Leiden, prothrombin gene polymorphism G20210A, elevated levels of factor VIII, and deficiencies of protein C, protein S, or antithrombin [6]. This patient was suspicious for a possible genetic prothrombotic condition due to her family history of cerebrovascular accidents (CVAs) and deep vein thrombosis (DVTs). Her prior genetic testing revealed a normal factor V Leiden genotype and no prothrombin gene polymorphisms, and outpatient labs revealed normal quantitative tests of factor VIII, protein C, protein S, and antithrombin. Because she had a very limited number of other known risk factors that could have led to her PVT, further investigation of a prothrombotic condition via her MTHFR polymorphism was warranted.

MTHFR (5,10-methylenetetrahydrofolate reductase) is located on chromosome one and is important for the metabolism of folate, which is necessary for methylation of DNA, RNA, and proteins. A polymorphism in the MTHFR gene can potentially cause decreased enzyme activity, and thus, decreased conversion of methionine to cysteine and 5,10-methylenetetrahydrofolate to 5-methyltetrahydrofolate [3], with resultant elevated homocysteine. Homocysteinemia is defined as a level greater than 15 micromol/L and is severely elevated if greater than 60 micromol/L [9].

There are two MTHFR genes, one inherited from each parent. One gene mutation results in a heterozygous individual, while two gene mutations result in a homozygous individual [9]. Out of the known 24 different MTHFR polymorphisms, the most common is C677T [9,10]. Approximately 20-40% of the Hispanic and Caucasian population are heterozygous for this polymorphism in the United States, while 8-20% are homozygous for this polymorphism in North America, Europe, and Australia [9]. The C677T variant is due to the substitution of alanine for valine on codon 222, resulting in decreased enzyme function [3]. Conversely, the second common MTHFR variant is A1298C, caused by missense glutamine to alanine substitution at codon 429. This polymorphism is believed to result in a non-thermolabile MTHFR molecule, consequently not affecting homocysteine levels [10], though data on the miscellaneous MTHFR variants and their effects is limited. In this case, our patient was found to be homozygous for MTHFR A1298C and did not have MTHFR C677T. Her homocysteine levels were normal at the time of her genetic testing but not quantified at the time of her PVT. Though MTHFR A1298C has not been shown to affect the enzyme’s function and subsequent homocysteine levels, research is conflicting regarding MTHFR’s possible association with a prothrombotic condition. Some studies found that the MTHFR A1298C polymorphism was significantly more common in patients with deep vein thrombosis than controls [11], while others found that neither MTHFR C677T nor A1298C was significantly associated with deep vein thrombosis [12], only MTHFR C677T is associated with thrombosis [13], or that either variant is associated with thrombosis but only when present with factor V Leiden [14]. Thus, further research is warranted to investigate the possible association of the different MTHFR variants with incidences of thromboses, with and without other genetic hypercoagulable states and risk factors, as well as in association with homocysteine levels. 

One limitation of this case is that the patient’s homocysteine levels were not evaluated at the time of her PVT. The most common theory is that MTHFR may provoke a hypercoagulable state by causing homocysteinemia, but research findings regarding this have been inconsistent as well [6]. Some data suggest that homocysteine does in fact cause a prothrombotic state, while other articles suggest that elevated homocysteine only indicates increased cardiovascular risk [4,9] and lowering homocysteine levels does not decrease the risk of venous thrombosis [9]. Furthermore, research that does support hyperhomocysteinemia increasing hypercoagulability has not discovered a precise mechanism. One study demonstrated that homocysteine upregulates monocyte chemoattractant protein-1 and interleukin 8, which alter the endothelial function and progress atherogenesis [15], while another study showed higher homocysteine levels were associated with more collagen accumulation in vascular smooth muscle cells [16]. Additional research on how homocysteine may impact the body’s coagulability pathways is needed.

Regardless of homocysteine levels or the presence of MTHFR polymorphism, the treatment of an acute PVT is the same. The patient should receive anticoagulation for at least three months and preferably for six months [6]. In the hospital, this patient was treated with unfractionated IV heparin immediately upon diagnosis. Prompt recognition of a PVT is critical, as the rate of vessel recanalization may drop from 69% to 25% if anticoagulants are initiated on the second-week symptom onset rather than the first [1]. Our patient was then transitioned from IV heparin to oral rivaroxaban to continue for three to six months. Alternatives to anticoagulation include thrombolytic therapy, but this is typically reserved for patients who fail initial anticoagulation treatment as it is less effective and carries a higher mortality rate [1]. Alternatively, if a patient has one or more prothrombotic disorders, providers should consider treatment with lifelong anticoagulation. If a patient has an MTHFR polymorphism without other established prothrombotic disorders, no evidence suggests that the patient requires any additional medications or supplements. 

Due to the inconsistency regarding hyperhomocysteinemia and venous thrombosis, as well as a lack of testing of her homocysteine levels at the time of her PVT, it is unclear whether the patient’s MTHFR polymorphism did, in fact, predispose our patient for PVT via hyperhomocysteinemia. Analyzing case studies such as this one and conducting more extensive testing in PVT patients without apparent causes may provide additional insight into this discrepancy. 

## Conclusions

PVT is a rare diagnosis in general, but particularly among individuals without cirrhosis. In these instances, it is important to investigate other etiologies of hypercoagulability. In this case, an MTHFR polymorphism was suspected as a risk factor for her PVT when other established inherited or acquired conditions were ruled out. Because data is conflicting regarding whether MTHFR prompts thrombosis and homocysteine levels were not quantified at the time of her diagnosis, one cannot conclude that the patient’s MTHFR polymorphism or a state of hyperhomocysteinemia caused her portal vein thrombosis. This case illustrates the need for future studies investigating different types of MTHFR polymorphisms, their potential effects on homocysteine levels, and how hyperhomocysteinemia may or may not contribute to hypercoagulability. Further research on MTHFR and its role in coagulation will help determine whether it may be beneficial for providers to test for MTHFR when evaluating risk factors for thrombosis and may help optimize treatment for patients found to have particular polymorphisms.
